# Supplementary material for: Resolving the full spectrum of human genome variation using Linked-Reads
Source: Genome Res. 2019 Apr;29(4):635–45. doi: 10.1101/gr.234443.118 (PMC6442396; doi:10.1101/gr.234443.118)
Supplement: Supplemental Material [file supp_gr.234443.118_Supplemental_longranger-2.2.zip › longranger-2.2.tar.bz2!/lr2.2-public/lib/stan/cmdstan/stan/doc/api/html/namespaceboost_1_1math.html]

Stan: boost::math Namespace Reference


|  |  |
| --- | --- |
|  | Stan  2.5.0  probability, sampling & optimization |


- Main Page
- Related Pages
- Namespaces
- Classes
- Files

- Namespace List
- Namespace Members

All Classes Namespaces Files Functions Variables Typedefs Enumerations Enumerator Friends Macros Pages

- boost
- math

Functions

boost::math Namespace Reference

Reimplmeneting boost functionality for `stan::agrad::var` and and bugs in classification of integer types.
More...

|  |  |
| --- | --- |
| Functions | |
| template<> | |
| int | fpclassify (const stan::agrad::var &v) |
|  | Categorizes the given stan::agrad::var value. More... |
|  | |
| template<> | |
| bool | isfinite (const stan::agrad::var &v) |
|  | Checks if the given number has finite value. More... |
|  | |
| template<> | |
| bool | isinf (const stan::agrad::var &v) |
|  | Checks if the given number is infinite. More... |
|  | |
| template<> | |
| bool | isnan (const stan::agrad::var &v) |
|  | Checks if the given number is NaN. More... |
|  | |
| template<> | |
| bool | isnormal (const stan::agrad::var &v) |
|  | Checks if the given number is normal. More... |
|  | |

## Detailed Description

Reimplmeneting boost functionality for `stan::agrad::var` and and bugs in classification of integer types.

FIXME: remove when BOOST fixes isfinite(). See ticket #6517. (Boost 1.48.0) https://svn.boost.org/trac/boost/ticket/6517

## Function Documentation

template<>

|  |  |  |  |  |  |
| --- | --- | --- | --- | --- | --- |
| int boost::math::fpclassify | ( | const stan::agrad::var & | *v* | ) |  |

inline

Categorizes the given stan::agrad::var value.

Categorizes the stan::agrad::var value, v, into the following categories: zero, subnormal, normal, infinite, or NAN.

Parameters
:   |  |  |
    | --- | --- |
    | v | Variable to classify. |

Returns
:   One of `FP_ZERO`, `FP_NORMAL`, `FP_FINITE`, `FP_INFINITE`, `FP_NAN`, or `FP_SUBZERO`, specifying the category of v.

Definition at line 24 of file boost\_fpclassify.hpp.

template<>

|  |  |  |  |  |  |
| --- | --- | --- | --- | --- | --- |
| bool boost::math::isfinite | ( | const stan::agrad::var & | *v* | ) |  |

inline

Checks if the given number has finite value.

Return `true` if the specified variable's value is finite.

Parameters
:   |  |  |
    | --- | --- |
    | v | Variable to test. |

Returns
:   `true` if variable is finite.

Definition at line 39 of file boost\_fpclassify.hpp.

template<>

|  |  |  |  |  |  |
| --- | --- | --- | --- | --- | --- |
| bool boost::math::isinf | ( | const stan::agrad::var & | *v* | ) |  |

inline

Checks if the given number is infinite.

Return `true` if the specified variable's value is infinite.

Parameters
:   |  |  |
    | --- | --- |
    | v | Variable to test. |

Returns
:   `true` if variable is infinite.

Definition at line 54 of file boost\_fpclassify.hpp.

template<>

|  |  |  |  |  |  |
| --- | --- | --- | --- | --- | --- |
| bool boost::math::isnan | ( | const stan::agrad::var & | *v* | ) |  |

inline

Checks if the given number is NaN.

Return `true` if the specified variable has a value that is NaN.

Parameters
:   |  |  |
    | --- | --- |
    | v | Variable to test. |

Returns
:   `true` if variable is NaN.

Definition at line 69 of file boost\_fpclassify.hpp.

template<>

|  |  |  |  |  |  |
| --- | --- | --- | --- | --- | --- |
| bool boost::math::isnormal | ( | const stan::agrad::var & | *v* | ) |  |

inline

Checks if the given number is normal.

Return `true` if the specified variable has a value that is normal.

Parameters
:   |  |  |
    | --- | --- |
    | v | Variable to test. |

Returns
:   `true` if variable is normal.

Definition at line 84 of file boost\_fpclassify.hpp.


---

[ Stan Home Page ]

*© 2011–2014,
Stan Development Team.*
